# Supplementary material for: Neogene amphibians and reptiles (Caudata, Anura, Gekkota, Lacertilia, and Testudines) from the south of Western Siberia, Russia, and Northeastern Kazakhstan
Source: PeerJ. 2017 Mar 23;5:e3025. doi: 10.7717/peerj.3025 (PMC5366065; doi:10.7717/peerj.3025)
Supplement: Supplemental Information 2 — The numbering of the localities corresponds to the locality numeration in the Figs. 1, 2, S1 and S4. [file peerj-05-3025-s002.docx]

**MIDDLE MIOCENE**

**Kalkaman Svita** (*Zykin & Zazhigin*, 2008; *Zykin*, 2012)

1. *Malyi Kalkaman 1*: *Heterosminthus* aff. *orientalis* and *Microtocricetus molassicus* are found from here (*Zykin & Zazhigin*, 2008). Association of *Dicrocerus elegans*, *Miotragocerus* sp., *Anchitherium aurelianense* and *Microtocricetus* cf. *molassicus* is unusual, since *D. elegans* is unknown in Europe younger than 13.5 Ma (*Böhme* et al., 2012) and *Microtocricetus molassicus* appears at 11.6 Ma (*Kirscher* et al., 2016). Here we very tentatively assume an age of about 12.5 Ma. Beside *M. molassicus*,. No palaeomagnetic data.
2. *Malyi Kalkaman 2*: *Microtocricetus molassicus*, fossiliferous horizon 4-5 m below the Malyi Kalkaman 1. No palaeomagnetic data.
3. *Baikadam*: upper part of Kalkaman svita, *Microtocricetus molassicus*, so presumably younger than Malyi Kalkaman 1. No palaeomagnetic data.

**LATE MIOCENE**

**No Svita**

1. *Shet-Irgyz 1*: *Heterosminthus mugodzharicus*, no palaeomagnetic data. 10-11 Ma, MN 9.

48. *Shet-Irgyz 2*: *Heterosminthus mugodzharicus*, no palaeomagnetic data. 10-11 Ma, MN 9.

**Ishym Svita** (*Zykin*, 2012)

1. *Petropavlovsk 1*: *Ishimomys quadriradicatus*, no palaeomagnetic data; early Turolian small mammals, correlated to Maeotian, age ca. 9-10 Ma.

**No** **Svita**

1. *Znamenka*: contains mixed fauna.

**Pavlodar Svita** (*Gnibitenko*, 2006; *Zykin*, 2012)

The section has 7 reversals attributed to C4n.1n to base C3Ar.

1. *Pavlodar 1A (=Gusiniy Perelet)***:** *Lophocricetus vinogradovi, Rhinocerodon pauli,* inverse polarity (correlated to C3Br.3r), Tortonian-Messinian boundary, age 7.25 Ma.

**Kedey Svita** (*Zazhigin*, 2006; *Zykin*, 2012)

Same small mammals as Pavlodar 1A, no palaeomagnetic data. 6.7-7.1 Ma.

1. *Selety 1A*: *Lophocricetus vinogradovi, Rhinocerodon seletyensis.*
2. *Kedey*: *Sibirosminthus seletyensis.*

**Novaya Stanitsa Svita** (*Gnibitenko*, 2006; *Zykin*, 2012)

1. *Nova Stanitsa 1A*: *Lophocricetus afanasievi, Sibirosminthus latidens, Prosomys* sp. The locality comes from the base Nova Stanitsa Svita, which has normal polarity (C3An.2n), age 6.6-6.5 Ma.
2. *Borki 1A*: base of the Nova Stanitsa Svita, no palaeomagnetic data.
3. *Lezhanka 2A*: base of the Nova Stanitsa Svita, no palaeomagnetic data.

**Rytov Svita** (*Gnibitenko*, 2006; *Zykin*, 2012)

1. *Cherlak*: the base of the Rytov Svite at Cherlak section has normal polarity. Upwards, the section is characterised by a long inverse polarity (C3r). The fossiliferous layer is situated at the base of the inverse chron. It contains *Lophoscircetus sibiricus*, *Sibirosminthus latidens*, *Prosomys* sp., *Plioscirtopoda rapida*. *Plioscirtopoda rapida* like Pavlodar 1B. Age 5.9-5.8 Ma.
2. *Pavlodar 1B*: 1 m of sands erosively overlaying the Pavlodar Svita. *Rhinocerodon irthyshensis*, *Sibirosminthus irtyshensis*, *Plioscirtopoda rapida.* No palaeomagnetic data.
3. *Lezhanka 2B*: No palaeomagnetic data.

**EARLY PLIOCENE**

**Iskakova Svita** (*Zykin & Zazhigin*, 2004; *Zykin*, 2012)

1. *Iskakova 2A*: *Promimomys insuliferus*, lithostratigraphically correlated to Iskakova 1A. No palaeomagnetic data.
2. *Iskakova 1A*: the sediment of the horizon shows inverse polarity and is situated below three normal ones (Gilbert) 🡪 top C3r. Age ~5.4-5.23 Ma.

**Peshniovo Svita** (*Zykin*, 2012)

1. *Peshniovo*: *Lophocricetus sibiricus*, *Prosomys antiquus*, normal polarity (C3n.3n). Age 4.896-4.799 Ma.

**Krytogorska Svita** (*Zykin & Zazhigin*, 2004; *Zykin*, 2012)

1. *Iskakova 1B*: *Prosomys* cf. *davakosi* normal polarity (C3n.2n). Age 4.631-4.493 Ma.

**No Svita**

1. *Olkhovka 1A*
2. *Olkhovka 1B*
3. *Olkhovka 1C*

For all these three localities no assignment to a svita is available. The fossil horizons lay between the Beteke and Rytov Svitas, small mammals fauna can be referred to MN14. Age 4.3-4.6 Ma. No palaeomagnetic data. (*Zykin*, 2012, fig. 4.17).

**No Svita**

1. *Kamyshlovo*: *Lophocricetus* *ultimus*, age 4.3-4.6 Ma.

**Beteke Svita** **(*Zykin*, 2012)**

The svita has inverse polarity in the lower half and normal polarity upper half of the section. 4.3-4.15 Ma.

1. *Beteke 1B.*
2. *Pavlodar 2B.*
3. *Pavlodar 3A.*

**Liven Svita** (*Zykin*, 2012)

The entire svita shows inverse (C2Ar) polarity. *Promimomys* *gracilis*, *Lophocricetus ultimus*, primitive *Mimomys* (more progressive than from Beteke 1B). Age 4.18-3.6 Ma.

1. *Lezhanka 1.*
2. *Andreievka-Speransko.*
3. *Livenka.*
4. *Lezhanka 1.*

**LATE PLIOCENE**

**Selety Svita** (*Zykin*, 2012)

1. *Beteke 1C*: the base of the svita has inverse polarity, the fossil fauna (*Mimomys polonicus* (MN16b)) comes from the part with normal polarity (C2An.1n). Age 3.03-2.8 Ma.

**EARLY PLEISTOCENE**

**Irthysh Svita** (*Zazhigin*, 2009; *Zykin*, 2012)

*Mimomys pliocaenicus* zone (MN17), late Villanian. Mainly inverse polarized (C2r.1r), the base of svita has normal polarization (Reunion). Age 2.0-2.14 Ma.

1. *Lebiazhie 1A.*
2. *Lebiazhie 1B.*
3. *Podpusk 1.*

(*Vdovin & Galkina*, 1976; *Zazhigin*, 2009)

1. *Kamen-na-Obi*: normal polarity, *Mimomys pliocaenicus* zone (MN17). Age ?~1.9 Ma

**Karagas Svita** (*Zazhigin*, 2009; *Zykin*, 2012)

The section of the 6m sands shows in the base normal polarity, the top has inverse polarity.

1. *Beteke 2*: *Allophaiomys pliocaenicus* zone (*Mimomys pusillus*), early Biharian; normal polarity. Age 1.8 Ma.
2. *Beteka 4:* fauna as Beteke 2, but inverse polarity. Age ~1.7 Ma.

**Kochkov Svita** (*Zazhigin*, 2009; *Zykin*, 2012)

1. *Razdole*: *Allophaiomys pliocaenicus* zone (*Mimomys pusillus*), inverse polarization. Age 1.25-1.78 Ma.

References

**Böhme** **M, Aiglstorfer** **M, Uhl** **D, Kullmer** **O. 2012.** The Antiquity of the Rhine River: Stratigraphic Coverage of the *Dinotheriensande* (Eppelsheim Formation) of the Mainz Basin (Germany). *PLoS ONE* **7 (5)**:e36817.

**Gnibitenko** **ZN. 2006.** *Cenozoic paleomagnetism of the West Siberian Plate*. Novosibirsk: Geo.

**Kirscher** **U, Prieto** **J, Bachtadse** **V, Abdul Aziz** **H, Doppler** **G, Hagmaier** **M, Böhme** **M. 2016.** A biochronologic tie-point for the base of the Tortonian stage in European terrestrial settings: Magnetostratigraphy of the topmost Upper Freshwater Molasse sediments of the North Alpine Foreland Basin in Bavaria (Germany). *Newsletters on Stratigraphy* **49 (3)**:445–467.

**Vdovin** **VB, Galkina** **LI. 1976.** Elements of Tiraspolian and Khaprovian Faunistic Complexes of the Antropogene in town Kamen-na Obi. In: Nikolaev VA, Kashmenskaya OV, eds. *Geomorphology and of the Quaternary Geology of the North Asia.* Novosibirsk: Nauka, 135–141.

**Zazhigin** **VS. 2006.** On small mammal fauna of arid zones from the late Miocene of the Southwestern Siberia. In: Matishov GG, ed.: SSC RAS Publishing hous, 198–202.

**Zazhigin** **VS. 2009.** Complexes of the small mammals of the late Pliocene - early Pleistocene of the south of the West Siberia. In: Lavrushin, Yu. A., Volkova, V. S., Khazina, I. V., ed. *Proceeding of the VI All-Russian Quaternary Conference.* Novosibirsk: Siberian dept. of Russian Academy of Sciences, 218–624.

**Zykin** **VS. 2012.** *Stratigraphy and evolution of environments and climate during Late Cenozoic in the Southern West Siberia*. Novosibirsk: Geo.

**Zykin** **VS, Zazhigin** **VS. 2004.** A new biostratigraphic level of the Pliocene in Western Siberia and the age of the Lower-Middle Miocene stratotype of the Beshcheul Horizon. *Doklady Earth Sciences* **398 (7)**:904–907.

**Zykin** **VS, Zazhigin** **VS. 2008.** On the Neogene stratigraphy of Pavlodarian Priirtyshya. In: Tleuberdina PA, Erzhanov NT, Zykin VS, eds.: Pavlodarian State University, 15–21.s
