# Supplementary material for: Neogene amphibians and reptiles (Caudata, Anura, Gekkota, Lacertilia, and Testudines) from the south of Western Siberia, Russia, and Northeastern Kazakhstan
Source: PeerJ. 2017 Mar 23;5:e3025. doi: 10.7717/peerj.3025 (PMC5366065; doi:10.7717/peerj.3025)
Supplement: Supplemental Information 3 — We provide also available assignments to stratigraphic horizons, ages, svitas, basins and literature sources. The locality numbering corresponds to the locality enumeration given in the Figs. 1 and 2, as well as in the Supplemental Information 1. [file peerj-05-3025-s003.docx]

**LATE OLIGOCENE**

1. **Akespe**, Aral Horizon, Upper Oligocene (Late Oligocene Biozone C and C1, *Daxner-Höck* et al. (2013), p. 491).

**Amphibia**

*Mioproteus* sp. IP Tiblisi (mentioned in *Malakhov* (2003), from the collection of the Institute of Zoology, Almaty, RK)

**Small mammals** (*Bendukidze*, 1993; *Bendukidze, Bruijn & Van den Hoek Ostende, Lars W.*, 2009)

*Amphechinus minimum*

*Eucricetodon (Atavocricetodon) occasionalis*

*Eucricetodon* aff. *caducus*

*Desmatolagus* aff. *shargaltensis*

*Desmatolagus* aff. *gobiensis*

*Tachyoryctoides* aff. *obrutschewi*

*Tachyoryctoides spurius*

*Aralomys gigas*

Bendukidze (1989) mentioned a vertebrate assemblage (amphibian and mammal) from Agyspe, representing mix of three different horizons (localities).

**EARLY MIOCENE**

1. **Ayakoz**, Early Miocene, Aquitanian, Zaysan Basin. The age based on the small mammal *Heterosmithus* *firmus* (*Zazhigin & Lopatin*, 2000), correlable with the Biozone D (20.4-22.5 Ma) of the Valley of Lakes, Mongolia see Daxner-Höck et al. (*Daxner-Höck, Böhme & Kossler*, 2013).

**Amphibia**

aff. *Tylototriton* sp. (coll. IZ Tbilisi)

(?) *Chelotriton* sp. (coll. IZ Tbilisi)

*Mioproteus* sp. (coll. IZ Tbilisi)

*Rana* sp. (coll. Uni. Prague)

*Bufotes* aff. *viridis* (*Malakhov*, 2005)

**Reptilia**

*Melanochelys* sp. (*Chkhikvadze*, 1989)

*Varanus* sp. (*Malakhov*, 2005)

*Eryx* sp. (*Malakhov*, 2005)

**Small mammals**

*Heterosmithus* *firmus* (*Zazhigin & Lopatin*, 2000)

*Synexalleris otus* (*Lopatin & Zazhigin*, 2003)

*Pseudotheridomys emry* (*Tyutkova*, 2009a)

*Rhodanomys bayshashovy* (*Tyutkova*, 2009a)

*Prodryomys* sp. (*Tyutkova*, 2009a)

*Agnotocastos* sp. (*Tyutkova*, 2009b)

*Stenofiber deperti* (*Tyutkova*, 2009b)

*Asiacastor antecedens* (*Tyutkova*, 2009b)

*Ayakosomys sergyopolis* (*Tyutkova*, 2009b)

*Aralomys padre* (*Tyutkova*, 2009b)

*Parasminthus tongingoli* (*Tyutkova*, 2009b)

*Parasminthus* sp. (*Tyutkova*, 2009b)

*Distylomys nata* (*Tyutkova*, 2009b)

*Prodistylomys* sp. (*Tyutkova*, 2009b)

*Yindirtemys minimus* (*Tyutkova*, 2009b)

*Sayimys* sp. (*Tyutkova*, 2009b)

*Plesiosciurus sinensis* (*Tyutkova*, 2009b)

*Sinotamias maximus* (*Tyutkova*, 2009b)

**Large mammals** (*Bayshashov*, 2001)

*Aprotodon ayakozensis*

*Protaceratherium* sp.

*Palaeochoerus* sp.

*Amphitragulus* sp.

1. **Golubye pesky**, Akzhar Svita, Zaysan Basin, Early Miocene (Burdigalian).

**Reptilia**

*Chelydropsis poena* (*Chkhikvadze*, 1989; p. 10)

*Pelodiscus* sp. (*Chkhikvadze*, 1989; p. 19) (cf. *Pelodiscus jakhimovitchae*, Kordikova (1994))

*Kazakhemys* *zaisanensis* (*Chkhikvadze*, 1989; p. 21)

*Baicalemys* *jegalloi* (*Chkhikvadze*, 1989; p. 23)

*Ocadia* *iliensis* (*Chkhikvadze*, 1989; p. 30)

**Small** **mammals**

*Sinolagomys pachygnathus* (*Erbaeva*, 1994; p. 69)

*Heterosminthus* *honestus* (*Zazhigin & Lopatin*, 2000; p. 98)

1. **Zmei Gorynych**, (probably Zmeinoe, River Cherniy Irtysh, *Heterosminthus honestus* (*Zazhigin & Lopatin*, 2000)), Akzhar Svita, Zaysan Basin, Early Miocene (Burdigalian). Profile (Nr. 19) of the Ashutas Mountain is given in (*Martinson & Kyansep-Romashkina*, 1980; fig. 2). This locality corresponds probably to the loc. Nr. "22. Ashutas" in (*Chkhikvadze*, 1973; p. 9).

**Amphibia**

Bufonidae indet. (*Chkhikvadze*, 1985)

**Reptilia**

*Anguis* sp. (*Chkhikvadze*, 1985)

*Pelias* sp. (*Chkhikvadze*, 1985)

*Vipera xanthina* (*Chkhikvadze*, 1985)

*Eryx* sp. (*Chkhikvadze*, 1985)

*Protestudo alba* (*Chkhikvadze*, 1989)

**Small mammals**

*Desmatolagus* sp. (*Erbaeva*, 1994; p. 70)

*Bellatona kazakhstanica* (*Erbaeva*, 1994; p. 70)

*Heterosminthus* *honestus* (*Zazhigin & Lopatin*, 2000; p. 98)

1. **Vympel**, South-Easter coast of Zaysan Lake, Akzhar Svita, Zaysan Basin, Early Miocene (Burdigalian).

**Amphibia**

*Andrias karelcapeki* (*Böhme, Vasilyan & Winklhofer*, 2012)

**Reptilia**

*Kazakhemys zaisanensis* (*Chkhikvadze*, 1989; p. 21)

*Baicalemys jegalloi* (*Chkhikvadze*, 1989; p. 23)

*Pelodiscus* sp. (*Kordikova*, 1994)

**Small mammals**

*Gobiolagus* sp. (*Chkhikvadze*, 1989; p. 69)

*Amphilagus* sp. (*Chkhikvadze*, 1989; p. 69)

*Sinolagomys pachygnathus* (*Chkhikvadze*, 1989; p. 69). The species is known from the terminal Oligocene of the Xining Basin (*Li & Qui*, 1980; *Dai* et al., 2006)

1. **Poltinik**, South-Easter coast of Zaysan Lake, Akzhar Svita, Zaysan Basin, Early Miocene (Burdigalian).

**Amphibia**

*Andrias karelcapeki* (*Böhme, Vasilyan & Winklhofer*, 2012)

**Small mammals**

*Sinolagomys* cf. *pachygnathus* (*Erbaeva*, 1994)

1. **Zaezd**, Akzhar Svita, Zaysan Basin, Early Miocene (Burdigalian).

**Reptilia**

*Pelodiscus jakhimovitchae* (*Chkhikvadze*, 1989; p. 17)

**Small mammals**

*Sinolagomys pachygnathus* (*Erbaeva*, 1994; p. 70)

1. **Tri Bogatyrja**, South-Easter coast of Zaysan Lake, Akzhar Svita, Zaysan Basin, Early Miocene (Burdigalian).

**Amphibia**

*Andrias karelcapeki*, 15.00–18.00 Ma (*Böhme, Vasilyan & Winklhofer*, 2012)

1. **Kaymanovaja cherepakha**, middle Akzhar Svita (*Chkhikvadze*, 1989; p. 10), Zaysan Basin, Early Miocene (Burdigalian).

**Reptilia**

*Chelydropsis* *poena* (*Chkhikvadze*, 1989; p. 10)

*Pelodiscus* sp. (*Chkhikvadze*, 1989; p.19)

*Ocadia* *iliensis* (*Chkhikvadze*, 1989; p. 30)

**MIDDLE MIOCENE**

1. **Ryzhaya II** (Ryzhaya Sopka), South-Easter coast of Zaysan Lake, Zaisan Svita, (?early) Middle Miocene (*Erbaeva*, 1994).

**Amphibia**

*Mioproteus* sp. (*Chkhikvadze*, 1984 and present work), material stored at the IP of Tbilis

**Small mammals**

*Alloptox* ex gr. *gobiensis* (*Erbaeva*, 1994)

1. **Kentyubek**, Turgay Depression Basin, green, green-gray clay with manganous- ferruginous concretions (*Bendukidze & Chkhikvadze*, 1976; *Chkhikvadze*, 1989; *Bendukidze*, 1993). Late Middle Miocene (pers. comm. of Jerome Prieto, based on *Dinosorex* cf. *pachygnathus*).

**Amphibia**

*Mioproteus caucasicus* ((*Chkhikvadze*, 1984) and present study) material stored at the IP of Tbilis

Ranidae indet. (coll. Uni. Prague) (*Böhme & Ilg*, 2003)

Bufonidae indet. (*Bendukidze & Chkhikvadze*, 1976)

**Reptilia**

(Bendukidze & Chkhikvadze 1976)

*?Chelydropsis* sp. (*Bendukidze & Chkhikvadze*, 1976)

*?Ocadia* sp. (*Bendukidze & Chkhikvadze*, 1976)

*Chrysemys* sp. (*Bendukidze & Chkhikvadze*, 1976)

*Emydoidae tasbaka* (syn. *Emys* sp. in *Bendukidze & Chkhikvadze* (1976)) (*Chkhikvadze*, 1989)

*Kazakhemys zaisanensis* (*Planiplastron* sp. in *Bendukidze & Chkhikvadze* (1976)) (*Chkhikvadze*, 1989)

**Small mammals** (*Bendukidze*, 1993)

*Migalea* sp.

*Migalinia* sp.

*Desmana kentjubensis*

*Desmanella* sp.

*Blarinella* sp.

*Dinosorex* cf. *pachignathus*

*Desmatolasug kazachstanicus*

*Bellatona* sp.

*Tamias* sp.

*Democricetodon* aff. *freisingensis*

*Tsaganocricetus turgaiensis*

*Pseudotheridomys* sp

*Trogontherium minutum*

*Asiacastor* cf. major

*Asiacastor* cf. *baschanovi*

1. **Ashut**, Turgay Depression, Turme Svita. Middle Miocene (*Malakhov*, 2003, 2004, 2005; *Tyutkova*, 2008).

**Amphibia**

*Mioproteus* cf. *caucasicus* (*Malakhov*, 2003)

*Bufo* sp. (*Malakhov*, 2004)

**Small mammals** (*Tyutkova*, 2008)

Soricidae indet.

Ochotonidae indet.

*Monosaulux* sp.

*Eutamias* sp.

*Ansomys* cf. *orientalis*

*Gobicricetodon natalia*

*Megacricetodon* sp.

Eomyidae indet.

**Large mammals** (*Tyutkova*, 2008)

*Anchitherium* sp.

*Stephanocemas* sp.

*Gomphotherium* sp.

*Dicroceros* sp.

1. **Point Y** (Rus. Точка Ы) (= loc. K-39), sandy sediments, Sarybulak Svita, Zaisan Basin, late Middle Miocene – early Late Miocene. Geological description see *Borisov* (1963: p. 63) and *Kowalski & Shevyreva* (1997: p. 201).

**Reptilia**

*Pelodiscus jakhimovitchae* (holotype) (*Chkhikvadze*, 1989; p. 17, *Kordikova*, 1994)

*Protest udo darewskii* (*Chkhikvadze*, 1989; p. 58)

**Small mammalia**

*Miodryomys* cf. *biradiculus* (*Kowalski & Shevyreva*, 1997)

*Amphilagus* sp. (*Erbaeva*, 1994; p. 71)

*Alloptox cf. gobiensis* (*Erbaeva*, 1994; p. 71)

1. **Sarybulak Svita**, Zaysan Basin, without any assignment to locality (*Chkhikvadze*, 1985), late Middle Miocene – Late Miocene according to Vangengeim et al. (*Vangengeim* et al., 1993), Middle Miocene, *Heterosminthus jucundus*, loc. Point J [Rus. Точка Й] (*Zazhigin & Lopatin*, 2000).

**Reptilia** (*Chkhikvadze*, 1985)

Agamidae indet

Lacertidae indet.

*Anguis* sp.

*Eryx* sp.

*Pelias* sp.

**LATE MIOCENE**

1. **Kalmakpai Svita** (47°27'46.35"N, 85°23'46.62"E) Zaysan Basin, without any assignment to locality, Late Miocene (*Chkhikvadze*, 1985), Late Miocene (*Vangengeim* et al., 1993), middle Turolian (MN12).

**Reptilia** (*Chkhikvadze*, 1985)

*Ophisaurus* sp. “small sized” (=? *Anguis* sp. “large sized”)

*Varanus* sp.

*Eryx* sp.

**Large mammals**

*Vormela* sp.

*Adcrocuta eximia*

*Hyaenictitherium hyaenoides orlovi*

*Martes palaeosinensis*

Melinae gen. indet.

*Plesiogulo crassa*

*lndarctos* sp.

*Machairodus kurten*

*Hipparion hippidiodus*

*Hipparion elegans*

*Chilotherium* sp.

*Sinotherium zaisanense*

*Cervavitus novorossiae*

*Procapreolus latifrons*

*Gazella dorcadoides*

*Tragocerus* sp.

*Palaeotragus* (*Yuorlovia*) *asiaticus*

*Samotherium* cf. *irtyshense*

1. **Karabastuz**, north-east shore of the Karabastuz Lake, Pavlodar Svita, Late Miocene, Semipalatin Priirtyshie. Middle Turolian (MN12) (*Kuznetsov*, 1982; *Tleuberdina*, 2005).

**Reptilia**

*Protestudo karabastusica* (*Kuznetsov*, 1982)

**Aves** (*Tleuberdina*, 2005; p. 30)

*Struthio* sp.

**Small mammals** (*Tleuberdina*, 2005; p. 30)

*Proochotona* sp.

**Large mammals** (*Tleuberdina*, 2005; p. 30)

*Adcrocuta eximia*

*Plesiogulo* sp.

Machairodontidae gen. indet.

Mustelidae gen. indet.

*Zygolophodon* sp.

*Dicerorhinus* sp.

*Palaeotragus* (*Yuorlovia*) sp.

*Samotherium irtyshense*?

*Pliocervus karabastusicus*

*Eostylocerus propria*

*Hipparion elegans*

*Hipparion* *longipes*

*Hipparion* sp.

1. **Kalmakpai**, layer 365 see (*Borisov*, 1963; p. 69), Karabulak Svita, Zaysan Basin, terminal Late Miocene (*Sotnikova*, 1992). Middle Turolian (MN12).

**Reptilie**

*Protestudo illiberalis* (holotype) (*Chkhikvadze*, 1989; p. 59)

**Large mammals** (*Sotnikova*, 1992)

*Vormela* sp.

*Martes* sp.

*Plesiogulo crassa*

*Adcrocuta eximia*

*Hyaenictitherium hyaenoides orlovi*

*Machairodus kurteni*

*Hipparion hippidiodus*

*Hipparion elegans*

*Chilotherium* sp.

*Sinotherium zaisanensis*

*Cervavitus* sp.

*Procapreolus* sp.

*Samotherium* sp.

*Paleotragus* (*Yuorlovia*) *asiaticus*

*Tragocerus* sp.

*Gazella dorcadoides*

1. **Petropavlovsk 1/2**, Late Miocene – Early Pliocene. It is unknown if this fossil came from Petropavlovsk 1 (MN11) or Petropavlovsk 2 (MN14).

**Amphibia**

*Mioproteus* sp.

**EARLY PLIOCENE**

1. **Detskaya Zheleznaja Doroga (engl. Children Railway).** Miocene-Pliocene boundary, no svita assignment (*Gaiduchenko*, 1984; *Gaiduchenko & Chkhikvadze*, 1985). According to *Gaiduchenko* (1984), the locality represents a four meter sequence of mainly fluvial sediments (3rd and 4th layers in *Gaiduchenko* (1984)), overlaying reddish-brownish marls (*Gaiduchenko*, 1984). These sands show signs of reworking (marl clasts, *Gaiduchenko* (1984)) and preserve a fauna including all groups of vertebrates, which we were not able to study. *Promimomys* sp. together with beavers, *Paracamelus* (beside other large mammals), *Struthio*, *Chelydropsis* and several fishes has been reported in the fauna (*Gaiduchenko*, 1984; *Gaiduchenko & Chkhikvadze*, 1985). The association of steppe elements (*Paracamelus, Struthio;* both co-occur in the Rytov Svita, see below) with animals living in streams suggest stratigraphic mixing of faunas from different environmental settings, as already recognized by *Gaiduchenko* (1984). The age of the fluvial fauna may be near the Miocene-Pliocene boundary. Below we represent the fauna from the fluvial sediments (3rd and 4th layers in *Gaiduchenko* (1984)).

**Teleostei**

*Esox* sp*.*

Cyprinidae indet.

*Silurus* sp*.*

**Amphibia**

Cryptobranchidae indet.

**Reptilia**

? *Sakya* sp.

*Chelydropsis kusnetzovi*

**Aves**

*Struthio* sp.

**Small mammals**

*Ochotona* sp.

*Trogontherium minus*

*Promimomys* sp.

**Large mammals**

*Vulpes* sp.

*Anancus* sp.

*Hipparion* sp.

Rhinocerotidae gen. indet.

*Paracamelus* cf. *praebactrianus*

*Cervavitus* sp.

*Tragoceros* sp.

*Gazell* sp.

References

**Bayshashov** **BU. 2001.** New data on ancient ungulates from the locality Ayakoz and their biostratigraphy. *Geology of Kazakhstan* **5-6**:140–147.

**Bendukidze** **OG. 1989.** New data on small mammal fauna from the locality Agyspe. In: Agadjanian AK, Dubrovo IA, Reshetov VY, eds. *Operative-informative proceedings of the first Pansovietic conference on palaeotheriology.* Moscow, 11–13.

**Bendukidze** **OG. 1993.** *Miocene small mammals from South-Western Kazakhstan and Turgay*. Tbilisi: Metsniereba.

**Bendukidze** **OG, Bruijn** **H** de, **Van den Hoek Ostende, Lars W. 2009.** A revision of Late Oligocene associations of small mammals from the Aral Formation (Kazakhstan) in the National Museum of Georgia, Tbilissi. *Palaeodiversity* **2**:343–377.

**Bendukidze** **OG, Chkhikvadze** **VM. 1976.** Preliminary results of study on fossil amphibians, reptiles and birds from Turgay and Ustyurt. *Bulletin of the Moscow Society of Naturalists, Geological Series* **51 (5)**:156.

**Böhme** **M, Ilg** **A. 2003.** fosFARbase. Available at www.wahre-staerke.com (accessed 1 December 2015).

**Böhme** **M, Vasilyan** **D, Winklhofer** **M. 2012.** Habitat tracking, range dynamics and palaeoclimatic significance of Eurasian giant salamanders (Cryptobranchidae) — indications for elevated Central Asian humidity during Cenozoic global warm periods. *Palaeogeography, Palaeoclimatology, Palaeoecology* **342–343**:64–72.

**Borisov** **BA. 1963.** Stratigraphy of upper Cretaceus and Paleogene-Neogene of Zaisan basin. *Transactions of Pansoviet scientific-research geological institute* **New Series, 94**:11–75.

**Chkhikvadze** **VM. 1973.** New data on the Tertiary turtles of Georgia. *Bulletin of the Academy of Sciences of Georgian SSR* **69 (3)**:745–748.

**Chkhikvadze** **VM. 1984.** Survey of the fossil urodelan and anuran amphibians from the USSR. *Izvestia Akademii Nauk Gruzinska SSR, Seria Biologitcheskaya* **10 (1)**:5–13.

**Chkhikvadze** **VM. 1985.** Preliminary results of studies on tertiary amphibians and squamate reptiles of the Zaisan Basin. In: Darevsky I, ed.: Nauka, 234–235.

**Chkhikvadze** **VM. 1989.** *Neogene turtles of USSR*. Tbilisi: Metsniereba.

**Dai** **S, Fang** **X, Dupont-Nivet** **G, Song** **C, Gao** **J, Krijgsman** **W, Langereis** **C, Zhang** **W. 2006.** Magnetostratigraphy of Cenozoic sediments from the Xining Basin: Tectonic implications for the northeastern Tibetan Plateau. *Journal of Geophysical Research: Solid Earth* **111 (B11)**.

**Daxner-Höck** **G, Böhme** **M, Kossler** **A. 2013.** New Data on Miocene Biostratigraphy and Paleoclimatology of Olkhon Island (Lake Baikal, Siberia). In: Wang X, J FL, Fortelius M, eds. *Fossil mammals of Asia: Neogene biostratigraphy and chronology.* New York: Columbia University Press, 508–517.

**Erbaeva** **MA. 1994.** Stratigraphical distribution of Lagomorpha (Mammalia) in Tertiary deposites of Zaisan Depression (eastern Kazakhstan). In: Sokolov VE, ed. *Palaeotheriology.* Moscow: Nauka, 65–78.

**Gaiduchenko** **LL. 1984.** On stratigraphy of Neogene sediments of the southmost Westsiberian Plain. In: Volkova VS, Kul'kova LA, eds. *Environment and ligfe at the boundaries of Cenozoic epochs in SIberia and Far East.* Novosibirsk: Nauka, 172–184.

**Gaiduchenko** **LL, Chkhikvadze** **VM. 1985.** First record of chelydrid turtle from the Neogene sediments of Pavlodarian Priirtyshya. *Geologiya i geofizika* **(1)**:116–118.

**Kordikova** **EG. 1994.** Review of fossil Trionychid localities in the Soviet Union. *Courier Forschungsinstitut Senckenberg* **173**:341–358.

**Kowalski** **K, Shevyreva** **NS. 1997.** Gliridae (Mammali: Rodentia) from the Miocene of the Zaisan Depression (Eastern Kazakhstan). *Acta zoologica cracoviensia* **40 (2)**:199–208.

**Kuznetsov** **VV. 1982.** A terrestrial Pliocene tortoise from Karabastuz. *Materials of History of Fauna and Flora of Kazakhstan* **8**:17–21.

**Li** **C, Qui** **Z. 1980.** Early Miocene mammalian fossils of the Xining Basin, Qinghai Province. *Vertebrata Palasiatica* **18 (3)**:198–209.

**Lopatin** **AV, Zazhigin** **VS. 2003.** New Brachyericinae (Erinaceidae, Insectivora, Mammalia) from the Oligocene and Miocene of Asia. *Paleontologicheskij Zhurnal* **(1)**:64–77.

**Malakhov** **DV. 2003.** The earliest known record of *Mioproteus* (Caudata; Proteidae) from the Middle Miocene of Central Kazakhstan. *Biota* **4 (1-2)**:67–72.

**Malakhov** **DV. 2004.** Toads (Anura, Bufonidae) from the Middle Miocene in the Turgay Depression (Central Kazakhstan). *Biota* **5 (1-2)**:41–46.

**Malakhov** **DV. 2005.** The early Miocene herpetofauna of Ayakoz (Eastern Kazakhstan). *Biota* **6 (1-2)**:29–35.

**Martinson** **GG, Kyansep-Romashkina** **NP, eds. 1980.** *Paleolimnology of Zaysan*. Leningrad: Nauka.

**Sotnikova** **MV. 1992.** Anew species of Machairodus from the late Miocene Kalmakpai locality in eastern Kazakhstan (USSR). *Annales Zoologici Fennici* **28**:361–369.

**Tleuberdina** **PA. 2005.** Main stages of development of vertebrates fauna in Cenozoic of Kazakhstan. *Transactions of Institute of Zoology* **49**:12–37.

**Tyutkova** **LA. 2008.** The middle Miocene rodents of the Ashut locality (Turgay depression). *New Mexico Museum of Natural History Bulletin* **44**:437–442.

**Tyutkova** **LA. 2009a.** Early Miocene Eomyidae and Gliridae from Ayakoz (Eastern Kazakhstan). *Transactions of Institute of Zoology* **Animal Ecology of Kazakhstan. 50**:47–52.

**Tyutkova** **LA. 2009b.** On some rodents from Early Miocene age fauna of Ayakoz (Eastern Kazakhstan). *Transactions of Institute of Zoology* **Animal Ecology of Kazakhstan. 50**:41–47.

**Vangengeim** **EA, Vislobokova** **I, Godina** **AY, Dmitrieva** **EL, Zhegallo** **VI, Sotnikova** **MV, Tleuberdina** **PA. 1993.** On the age of mammalian fauna from the Karabulak Formation of the Kalmakpai River (Zaisan Depression, Eastern Kazakhstan). *Stratigraphy and Geological Correlation* **1 (2)**:38–45.

**Zazhigin** **VS, Lopatin** **AV. 2000.** The history of the Dipodoidea (Rodentia, Mammalia) in the Miocene of Asia: 1. *Heterosmithus* (Lophocricetinae). *Paleontologicheskij Zhurnal* **34 (3)**:90–102.
